# Praziquantel efficacy against *Schistosoma mansoni* among HIV-1 infected and uninfected adults living in fishing villages along Lake Victoria, Northwest Tanzania

**DOI:** 10.1186/2049-9957-3-47

**Published:** 2014-12-15

**Authors:** Humphrey D Mazigo, David W Dunne, Safari M Kinung’hi, Fred Nuwaha

**Affiliations:** Department of Medical Parasitology and Entomology, School of Medicine, Catholic University of Health and Allied Sciences, P.O. Box 1464, Mwanza, Tanzania; Department of Disease Control and Environmental Health, School of Public Health, College of Health Sciences, Makerere University, P.O. Box 7072, Kampala, Uganda; Department of Pathology, Division of Microbiology and Parasitology, Cambridge University, Tennis Court Road, Cambridge, CB2 1QP UK; National Institute for Medical Research, Mwanza Research Centre, P.O. Box 1462, Mwanza, Tanzania

**Keywords:** HIV-1, *S. mansoni*, Co-infection, Praziquantel, Efficacy, Tanzania

## Abstract

**Background:**

Animal studies have demonstrated that functional immune responses, as determined by the levels of CD4^+^ cell counts and anti-schistosome antibodies responses, determine the efficacy of praziquantel. Based on this evidence, it has been hypothesised that the immunodeficiency effects of the human immunodeficiency virus (HIV)-1 infection may affect the efficacy of praziquantel in co-infected human hosts. Thus, the present study assessed the efficacy of praziquantel by comparing parasitological cure rates and the reduction in infection intensity in HIV-1 seronegative individuals infected with *S. mansoni* and HIV-1 seropositive individuals co-infected with *S. mansoni,* following treatment with a single oral dose of praziquantel.

**Methods:**

This was a prospective longitudinal study which included, at baseline, 555 *S. mansoni* infected adults aged 21–55 years, who were either co-infected or not with HIV-1 and who lived in fishing villages along Lake Victoria in Northwest Tanzania. These individuals were treated with a single oral dose of praziquantel (40 mg/kg) and, at 12 weeks, single stool samples were obtained and examined for *S. mansoni* eggs using the Kato-Katz technique. Finger prick and venous blood samples were collected for HIV-1 screening and CD4^+^ cell quantification.

**Results:**

The parasitological cure rate did not differ significantly from the HIV-1 serostatus (*P* = 0.12): among the co-infected individuals, the cure rate was 48.3% (14/29), and among the individuals infected only with *S. mansoni*, the cure rate was 62.6% (329/526). The egg reduction rate did not vary with the HIV-1 serostatus (*P* = 0.22): 77.22% for HIV-1 seronegative and 75% for HIV-1 seropositive individuals. The level of CD4^+^ cell counts (median 228 cells/μL: range 202–380 cells) did not influence the cure rate (*P* = 0.23) or the reduction in the intensity of the infection (*P* = 0.37).

**Conclusion:**

The HIV-1 infection *per se* or its moderate immunodeficiency effects, demonstrated by the range of CD4^+^ cell counts observed in co-infected individuals, did not affect praziquantel efficacy, as measured by the parasitological cure rate and the reduction in intensity of infection in the present study cohort.

**Electronic supplementary material:**

The online version of this article (doi:10.1186/2049-9957-3-47) contains supplementary material, which is available to authorized users.

## Multilingual abstracts

Please see Additional file 1 for translations of the abstract into the six official working languages of the United Nations.

## Background

The World Health Organization (WHO) recommends the use of praziquantel for the treatment and morbidity control of schistosomiasis in endemic areas [1–3]. The drug has minimal serious side effects, is easy to administer and its price has been substantially reduced since its introduction [4–6]. Using a recommended single dose (40 mg/kg), the parasitological cure rates of praziquantel for *S. mansoni* ranges from 18% to 90% [7–11]. The reduction of the infection intensity reportedly ranges from 80% to 95%, when using the recommended single oral dose of 40 mg/kg and as assessed between four and 12 weeks after treatment [8, 12, 13].

The efficacy of praziquantel appears to depend on the synergy with the host’s intact immunological response against the adult worms [14–17]. Studies involving *S. mansoni* animal models have demonstrated the immune-dependent action of the drug, whereby immunodeficient animal models have a reduced efficacy [14–17]. Pre-immunisation of *S. mansoni* animals models with parasite antigens to generate parasite-specific antibodies [18, 19], or the passive transfer of immune serum from immunocompetent animal models, increases praziquantel efficacy [17, 19, 20]. This indicates that a functional and intact immune response is required to enhance praziquantel efficacy [19].

The demonstration that praziquantel efficacy is immune-dependent in *S. mansoni* animal models [16, 19, 20] suggests that human immunodeficiency virus (HIV)-1 associated immunodeficiency may affect praziquantel efficacy in human populations [21]. Two studies conducted in Western Kenya [22] and in rural Zimbabwe [23] found no significant difference in praziquantel efficacy in *S. mansoni* HIV-1 seronegative or seropositive individuals, in terms of cure rates or in reduction in intensity of infection, nor was there any influence of the CD4^+^ level on the treatment outcome [22, 23].

For over three decades, praziquantel has been the drug of choice for the treatment and control of the *S. mansoni* infection and morbidity [4], but there are concerns that the parasite may develop resistance against the drug [24]. Thus, the WHO highly recommends monitoring praziquantel efficacy in areas with dissimilar levels of *S. mansoni* endemicity [25, 26] and in infected individuals of differing immunological statuses [3]. Thus, it remains important to understand praziquantel efficacy in individuals concurrently infected with HIV-1 and *S. mansoni*, and with different immunological statuses (as measured by CD4^+^ levels). In the current study, we hypothesised that individuals co-infected with HIV-1 and *S. mansoni* have lower parasitological cure rates and praziquantel efficacy depending on their immune status as measured by CD4^+^ cell counts. We compared parasitological cure rates and infection intensity reductions after a single oral dose of praziquantel was given to HIV-1 seronegative and seropositive individuals infected with *S. mansoni.*

## Methods

### Study population and study area

The study was conducted in the Ilemela District, Mwanza Region, Northwest Tanzania. The study area is located at 32–34°E and 2–4°S, on the southern shores of Lake Victoria. Temperature range from 18°C to 28°C and there is a mean annual rainfall of 1068 mm. Four villages – Sangabuye, Kayenze, Igalagala and Igombe – were chosen for their close proximity to the lake. The majority of the population belongs to the Sukuma tribe. Other migrant tribes are the Kerewe, Jita and Kara. Most of the villagers in the four villages depend on the lake for domestic and economic activities. Domestic activities include washing, bathing, cooking, drinking and recreation, and the main economic activities are fishing and farming. Due to high water contact levels, residents have a high risk of being infected with *S. mansoni* [27–29], and a high occupational exposure maintains high intensities of *S. mansoni* infection into adulthood [30]. Annual mass drug administration (MDA) against helminth infections in these villages focuses on school-age children rather than the adult population. Moreover, the high rate of sexual mixing within the fishing villages increases the risk of HIV-1 transmission in the adult population [31]. In 2003, the HIV-1 prevalence in individuals aged 15–60 years living in Kayenze and Sangabuye villages was estimated to be 10% [27].

### Study design and sample size

The study consisted of a cross-sectional baseline study followed by a prospective interventional longitudinal follow-up. At baseline, the cross-sectional study was conducted to collect pre-treatment prevalence rates and intensities of the *S. mansoni* and HIV-1 infections. Following this, all infected participants – irrespective of their HIV-1 serostatus or CD4^+^ count levels – were treated with a single dose of praziquantel (40 mg/kg), and followed up after 12 weeks to assess the efficacy of the treatment. The study included individuals who had lived in the study villages for more than two years, either male or female, and aged from 21 to 55 years. Individuals with a history of treatment for schistosomiasis (praziquantel) in the past six months and those who were on antiretroviral treatment (ART) were excluded from the study at the baseline.

To calculate the sample size, we assumed a parasitological cure of 70% in HIV-1 negative individuals infected with *S. mansoni*. At a power of 80% and 95% confidence intervals to detect a parasitological cure rate of 60% among the HIV-1 positive individuals co-infected with *S. mansoni*, 33 HIV-1 positive individuals and 484 HIV-1 negative individuals would need to be treated with praziquantel, assuming a loss to follow-up of 10% and a prevalence of HIV among people infected with schistosomiasis of 6%. This sample size was estimated using Stata 12 with the command: sampsi 0.70 0.60, alpha (.95) power (.80) ratio (1.5).

### Data collection

(i) ***HIV-1 screening and CD4***^***+***^***analysis:*** All participants who were involved in the prospective longitudinal survey and were HIV-1 negative at the baseline cross-sectional survey were re-tested for the possibility of HIV-1 seroconversion according to the Tanzanian national HIV algorithms which use Determine® (Alere Determine, Chiba, Japan), followed by UNI-GOLD® (Trinity Biotech PLC, Bray, Ireland).
(ii) ***Parasitological examination of the S. mansoni infection:*** A single stool sample was obtained, from which four Kato-Katz thick smears were prepared [32] using a template of 41.7 mg (Vestergaard Frandsen, Lausanne, Switzerland). After 24 hours, the smears were independently examined for *S. mansoni* eggs by two experienced technicians from the National Institute for Medical Research (NIMR) laboratory. For quality assurance, a random sample of 10% of the negative and positive Kato-Katz thick smears were re-examined by a third technician.
(iii) ***Treatment and follow-up:*** All 854 individuals found to be infected with *S. mansoni* at baseline (aged 21–55 years), irrespective of their HIV-1 serostatus or CD4^+^ cell count levels, were treated with a single dose of praziquantel (40 mg/kg). These individuals were followed up and examined for *S. mansoni* eggs in their stool samples 12 weeks after treatment.

### Data analysis

The data were double entered using CSPro and the final data set was stored in a MySQL database. The data analysis was performed using Stata version 12 (StataCorp, College Station, Texas, USA). The parasitological cure rates and egg reduction of single-dose praziquantel was calculated only for individuals who gave single stool samples at baseline and at the follow-up examination 12 weeks later, from which four Kato Katz thick smears were prepared. In addition, these individuals received praziquantel treatment. The *S. mansoni* egg counts for each participant were calculated from the average of the counts from the four Kato-Katz thick smears and multiplied by 24 to obtain individuals’ eggs per gram of faeces (epg). To compare the groups, the geometric mean epg of the *S. mansoni* infection was obtained as the antilog of the mean of the transformed log (log epg + 1) egg counts of individuals in each group. The parasitological cure rates were calculated as the proportion of individuals who were excreting *S. mansoni* eggs in their stool samples prior to praziquantel administration and those who excreted no eggs after treatment, multiplied by a factor of 100. Egg count reduction rates were calculated as [1-(geometric mean epg after treatment/geometric mean epg before)] multiplied by a factor of 100. The parasitological cure rates were compared between demographic factors, HIV-1 serostatus; CD4^+^ cells count levels and the intensity of the *S. mansoni* infection. The geometrical mean epg at baseline and 12 weeks after treatment were compared with demographic factors, HIV-1 serostatus, CD4^+^ cells counts levels and the intensity of the *S. mansoni* infection using the t-test or ANOVA. Egg reduction rates at baseline and 12 weeks after treatment were compared using the χ^2^-test or Fisher’s exact test, where appropriate. The backward elimination logistic model was fitted to determine the factors associated with praziquantel treatment failure.

### Ethical considerations

Ethical approval was obtained from the Higher Degrees Research and Ethics Committee of the School of Public Health, Makerere University (IRB-00005856/2011), and from the joint CUHAS-Bugando Research and Publications Committee (BREC/001/32/2011). Ethical clearance was granted by the National Ethical Review Committee, National Institute for Medical Research, Tanzania, and the study was registered in the clinical trial network (Clinical Trial Number NCT-01541631).

The study received further authorisation from the regional and district administrative authorities of the Mwanza Region and Ilemela District. Swahili-translated informed assent and consent forms were used to obtain participants’ consent. For illiterate individuals, a thumbprint was used to sign the consent forms after a clear description of the study objectives was given.

All study participants who were infected with *S. mansoni* were treated with praziquantel (40 mg/kg) according to the WHO guidelines, irrespective of their HIV-1 serostatus. The study participants were counselled before and after HIV testing using the guidelines for HIV testing and counselling provided by the Ministry of Health and Social Welfare in Tanzania [33, 34]. Trained counsellors, working with the local district health departments, were involved in counselling the study participants before the HIV testing was done. All HIV infected individuals were given access to CD4^+^ counting, and those found to have CD4^+^ < 350 cells/μL were referred to the care and treatment clinic (CTC) at the nearby health centre and were then referred to the district hospital for further assessment of their eligibility for ART and management, according to the guidelines of the Ministry of Health and Social Welfare.

## Results

### Parasitological cure rates

**Figure 1:**
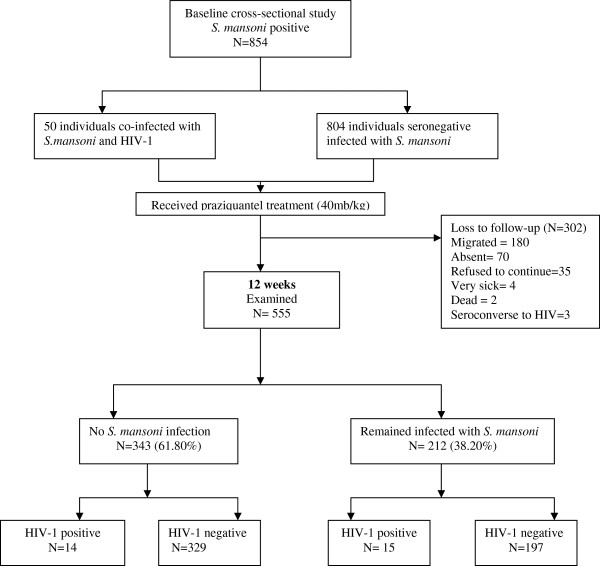
**Study profile showing** ***S. mansoni*** **infection among HIV-1 seropositive and seronegative at baseline and at 12 weeks follow-up point.**

Of the 854 individuals found infected at baseline and treated with praziquantel, 558 (65.33%) were present at the examination 12 weeks later. Three (3) individuals experienced HIV-1 seroconversion and were excluded from the analysis and 299 were absent due to various reasons, as shown in Figure 1. Pre-treatment parasitological data and demographic information were compared between individuals who were present and absent at the 12 weeks’ follow-up appointment. There were no differences in pre-treatment GM-epg between the 555 individuals who were present (174.21 epg, 95% CI; 154.14–196.87) and the 299 individuals who were absent (200.92 epg, 95% CI: 167.97–240.34) (t = −1.4012, *P* = 0.16). Similarly, there were no significant differences in sex distribution (χ^2^ = 0.9767, *P* = 0.32), age distribution (χ^2^ = 3.1831, *P* = 0.36), occupation (χ^2^ = 0.1168, *P* = 0.94) and village of residence (χ^2^ = 2.6712, *P* = 0.44).

The 555 individuals infected with *S. mansoni* at baseline gave a single stool sample at the 12 weeks’ follow-up examination. Of these, 526 were HIV-1 negative and infected with *S. mansoni* and 29 were co-infected at baseline. Of the co-infected individuals, 17 had a CD4^+^ cell count < 350cells/μL and 12 had a CD4^+^ count ≥ 350 cells/μL. The overall median CD4^+^ cell count was 228 cells/μL (range: 202–380).

The overall parasitological cure rate of the entire cohort was 61.80% (n = 343/555) after 12 weeks. In relation to the HIV-1 serostatus, among the 526 individuals of the HIV-1 negative cohort infected with *S. mansoni* at baseline, the parasitological cure rate was 62.55% (329/526) and, in individuals who were co-infected with HIV-1 and *S. mansoni,* the cure rate was 48.28% (14/29). Parasitological cure rates did not differ between the HIV-1 serostatus (*P* = 0.12). In relation to CD4^+^ count levels, the parasitological cure rate among HIV-1 and *S. mansoni* co-infected individuals with CD4^+^ cell counts <350 cells/μL was 43.75% (7/16) and those who had CD4^+^ cells counts of ≥ 350 cells/μL was 66.67% (8/12). However, the parasitological cure rates did not vary with the CD4^+^ count levels (*P* = 0.23). Table 1 shows the parasitological cure rates of praziquantel treatment in relation to demographic factors, HIV-1 serostatus, CD4^+^ cell count levels and the intensity of the *S. mansoni* infection. In general, the parasitological cure rates did not vary significantly in relation to HIV-1 serostatus, CD4^+^ cells counts levels and the intensity of the *S. mansoni* infection.

**Table 1:** **Parasitological cure rate of praziquantel treatment in relation to demographic factors, HIV-1, CD4** ^**+**^ **count levels and** ***S. mansoni*** **infection intensities**

*Variable*	*No. of individuals infected at baseline*	*No. of individuals cured after treatment (%)*	*χ* ^*2*^	P-value*
**Overall**	555	343 (61.80)		
**Sex**				
Female	241	148 (62.10)	0.028	0.87
Male	314	195 (62.10)		
**Age group**				
21–30	241	149 (61.83)	3.5550	0.31
31–40	177	103 (58.19)		
41–50	84	59 (70.24)		
51–60	53	32 (60.38)		
**Occupation**				
Small-scale business	66	38 (57.58)	0.5780	0.75
Farming	376	235 (62.50)		
Fishing	113	70 (61.95)		
**Village of residence**				
Igombe	112	67 (59.82)	3.0418	0.39
Igalagala	98	54 (55.10)		
Kayenze	219	140 (63.93)		
Sangabuye	126	82 (65.08)		
**Time of residence (years)**				
3–5	97	65 (67.01)	1.8745	0.59
6–10	83	53 (63.86)		
11–20	136	83 (61.03)		
≥21	239	142 (59.41)		
**HIV-1 serostatus**				
Negative	526	329 (62.55)	2.3714	0.12
Positive	29	14 (48.28)		
**CD4** ^**+**^ **count levels (cells/μL)**				
<350	17	7 (43.75)	1.4479	0.23
>350	12	8 (66.67)		
***S. mansoni*** **infection intensities**				
Light (1–100 epg)	234	143 (61.11)	0.9542	0.62
Moderate (101–399 epg)	162	105 (64.81)		
Heavy (≥400 epg)	159	95 (59.75)		

*P-value by chi-square (χ^2^).

### Effects of praziquantel treatment on S. mansoni infection intensities

The overall geometrical mean egg per gram of faeces (GM-epg) of the entire cohort of 555 individuals at baseline was 174.21 epg (95% CI: 154.14–196.87) and, after 12 weeks of treatment, the GM-epg was reduced to 39.88 epg (95% CI: 36.77–43.25). The overall egg reduction rate (ERR) for the entire cohort was 77.11%. In relation to the HIV-1 serostatus, at baseline, 526 HIV-1 seronegative individuals infected with *S. mansoni* had GM-epg of 175.06 epg (95% CI: 154.48–198.39), which decreased to 39.88 epg (95% CI: 36.69–43.55) in the 212 individuals who remained *S. mansoni* egg positive. This translated to an ERR of 77.22% after 12 weeks of treatment. For the co-infected individuals, at baseline, they had a GM-epg of 159.39 epg (95% CI: 27.20–58.11), which decreased to 39.85 epg (95% CI: 27.20–58.11) in the 15 co-infected individuals who remained *S. mansoni* egg positive at 12 weeks. This gave an ERR of 75%. The overall egg reduction rates did not differ with the HIV-1 serostatus (*P* = 0.22) (see Table 2). In relation to CD4^+^ count levels, at baseline, individuals with CD4^+^ cell count levels < 350 cell/μL had a GM-epg of 134.59 epg (95% CI: 52.19–347.07), which decreased to 49.66 epg (27.16–90.79), giving an ERR of 63.11%. In those who had CD4^+^ cell counts ≥ 350 cell/μL at baseline, the GM-epg was 215.78 epg (80.79–576.36), which decreased to 31.59 epg (95% CI: 13.18–75.69) after 12 weeks of treatment. For this group, the ERR was 85.37%. The ERR did not differ with the categories of CD4^+^ cell counts (*P* = 0.37). In relation to demographic factors, a significant ERR was observed in relation to age groups, with the younger age group (21–30 years) having an ERR of 81.11% (*P* < 0.05). Similarly, a significant ERR was observed in relation to the intensity of the *S. mansoni* infection (*P* < 0.0001), with individuals who had a heavy infection at baseline attaining a ERR of 95.19%. Table 2 shows the comparison of *S. mansoni* GM-epg at baseline and at the examination 12 weeks later, after treatment was stratified by demographic characteristics, HIV-1 serostatus, CD4^+^ cell count levels and intensity of the *S. mansoni* infection.

**Table 2:** ***Schistosoma mansoni*** **geometrical mean egg per gram of faeces (GM-epg) after praziquantel treatment in relation to demographic factors and** ***S. mansoni*** **infection intensities**

*Variable*	*Baseline before treatment*				*12 weeks after treatment*			
	N	GM-epg	95% CI	*P*-values	N	GM-epg	95% CI	*P*-values
**Overall**	555	174.21	154.14–196.87		212	39.88	36.77–43.25	
**Sex**								
Female	241	130.36	110.60–153.66	0.0001*	119	128.19	99.33–165.43	0.01
Male	314	217.41	183.01–258.29		93	222.37	168.53–293.41	
**Age group**								
21–30	241	198.67	163.49–241.42	0.25**	92	37.53	33.07–42.58	0.36**
31–40	177	156.18	126.12–193.41		74	43.95	38.14–50.66	
41–50	84	164.09	125.66–214.26		25	33.63	28.48–39.71	
51–60	53	152.13	98.56–234.79		21	45.30	33.83–60.66	
**Occupation**								
Small-scale business	66	136.05	99.65–185.74	0.08**	27	35.81	28.97–44.26	0.89**
Farming	376	157.92	135.73–183.74		141	39.58	35.71–43.88	
Fishing	113	278.74	214.63–362.00		44	43.65	36.72-51.88	
**Village of residence**								
Igombe	112	139.77	109.22–178.86	0.09**	45	36.28	31.16–42.24	0.38**
Igalagala	98	215.61	158.21–293.83		45	42.51	35.35–51.12	
Kayenze	219	197.91	162.96–240.35		78	41.12	35.58–47.52	
Sangabuye	126	144.04	110.22–188.24		44	38.99	32.51–46.76	
**Time of residence (years)**								
3–5	97	188.36	142.36–249.22	0.43**	33	43.18	34.74–53.67	0.66**
6–10	83	156.69	113.85–215.65		30	35.88	29.56–43.54	
11–20	136	204.77	159.53–262.83		52	41.16	35.20–48.14	
≥21	239	159.82	132.07–193.39		97	39.44	34.68–44.84	
**HIV-1 serostatus**								
Negative	526	175.06	154.48–198.39	0.56*	197	39.88	36.69–43.35	0.22*
Positive	29	159.39	86.09–295.14		15	39.85	27.20–58.41	
**CD4** ^**+**^ **count levels (cells/μL)**								
<350	17	134.59	52.19–347.07	0.75*	11	49.66	27.16–90.79	0.16*
>350	12	215.78	80.79–576.36		4	31.59	13.18–75.69	
***Schistosoma mansoni*** **infection intensities (epg)**								
Light (1–100 epg)	234	44.43	41.36–47.72	0.0001**	92	30.89	28.49–33.49	0.0001**
Moderate (101–399 epg	162	199.39	188.32–211.12		56	42.85	37.47–49.01	
Heavy (≥400 epg)	159	1124.33	987.62–1277.36		64	54.07	44.81–65.25	

*P-*values= t-test* and ANOVA**.

### Factors associated with praziquantel cure failure

Table 3 shows the bivariate and multivariable analysis, illustrating the factors associated with praziquantel cure failure. Neither at bivariate levels (OR = 1.79, 95% CI: 0.85–3.78, *P* = 0.13) nor at the multivariable analysis (aOR = 1.72, 95% CI: 0.69–1.39, *P* = 0.89) was HIV-1 infection *per se* associated with praziquantel treatment failure.

**Table 3:** **Factors associated with praziquantel cure failure**

*Variable*	*OR*	*95% CI*	*P-values*	*aOR*	*95% CI*	*P-values*
**Sex**						
Female	1		0.87	1		
Male	0.97	0.69–1.37		0.98	0.68–1.39	0.89
**Age group**						
21–30	0.94	0.51–1.73	0.84	0.93	0.50–1.71	0.81
31–40	1.09	0.59–2.05	0.78	1.08	0.58–2.02	0.82
41–50	0.65	0.31–1.33	0.24	0.65	0.31–1.34	0.24
51–60	1			1		
**Occupation**						
SME*	1			----	----	-----
Peasants	0.81	0.48–1.38	0.45	----	----	-----
Fishing	0.83	0.45–1.55	0.56	----	----	-----
**Village of residence**						
Igombe	1			----	----	-----
Igalagala	1.21	0.70–2.10	0.49	----	----	-----
Kayenze	0.84	0.53–1.34	0.47	----	----	-----
Sangabuye	0.79	0.47–1.35	0.40	----	----	-----
**HIV-1 serostatus**						
Negative	1			1		
Positive	1.79	0.85–3.78	0.13	1.72	0.69–1.39	0.89
**CD4+ counts (cells/μL)**						
<350	1			----	----	-----
≥350	0.38	0.08–1.84	0.23	----	----	-----
***S. mansoni*** **intensity of infection**						
Light	1			----	----	-----
Moderate	0.85	0.56–1.29	0.45	----	----	-----
Heavy	1.06	0.70–1.59	0.79	----	----	-----

*SME-Small business scale, OR = Odd Ratio aOR = Adjusted Odd Ratio.

## Discussion

In the present study, a relatively small percentage of the study population was co-infected with HIV-1 and *S. mansoni*, resulting in a low to moderate reduction in the CD4^+^ cell counts. Praziquantel efficacy, as measured by parasitological cure rates and reduction in the intensity of the infection, did not vary significantly with the HIV-1 serostatus and was not influenced by the level of CD4^+^ cell counts. In relation to the parasitological cure rate and the reduction in the intensity of infection, individuals co-infected with HIV-1 and *S. mansoni* responded to praziquantel treatment the same as HIV-1 seronegative individuals infected with *S. mansoni* did. Although co-infected individuals had lower cure rates and reduction in intensity of infection than the HIV-1 seronegative individuals infected with *S. mansoni*, the difference was not significant. Similarly, praziquantel efficacy – as measured by parasitological cure rates and the reduction in intensity of the infection – was not influenced by the level of CD4^+^ cell counts. The difference observed in parasitological cure rates and in reduction in intensity of infection in the two categories of CD4^+^ cell counts did not reach a significant level.

This observed praziquantel efficacy was consistent with previous studies conducted among humans co-infected with HIV-1 and *S. mansoni* [22, 23]. In rural Zimbabwe, the parasitological cure rates assessed 12 weeks after praziquantel treatment among individuals co-infected with HIV-1 and *S. mansoni* infection and those who were only infected with *S. mansoni* were 86% and 85%, respectively [23]. Equally, co-infected individuals and those only infected with *S. mansoni* experienced similar reductions in the intensity of the infection [23]. It should be noted that the Zimbabwean study was conducted in areas with low intensities of the *S. mansoni* infection [23]; this could partly explain the very high parasitological cure rates reported following treatment with a single dose of praziquantel [8, 12, 35]. A similar study was conducted in Western Kenya, in an area highly endemic for *S. mansoni* infection, among individuals with a heavy intensity of infection [22]. At four weeks following treatment, a parasitological cure rate of 59% in the HIV-1 seronegative individuals infected with *S. mansoni* and a rate of 53% among co-infected individuals were observed; an overall egg reduction of 93% was observed in the two groups [22]. Our study and the one done by Karanja *et al.* [22] were conducted among individuals highly infected with *S. mansoni,* based on egg counts. This may have in part contributed to the observed low parasitological cure rates. Previous studies have attributed a heavy intensity of *S. mansoni* infection, an intense transmission of infection and a low sensitivity to praziquantel among the strains of *S. mansoni* to low parasitological cure rates [7].

In the present study, praziquantel efficacy – as measured by the cure rate and reduced egg excretion – was not significantly influenced by the level of CD4^+^ cell counts, although individuals categorised as having a lower CD4^+^ cell count (<350 cells/μL) showed lower parasitological cure rates and egg excretion. Our observations were in accordance with studies conducted in HIV-1 co-infected individuals with low [23] and heavy intensities of infection [22]. In Western Kenya and rural Zimbabwe, egg reduction – assessed at four or 12 weeks after praziquantel treatment – did not differ over a range of CD4^+^ cell counts [22]. Our results on praziquantel efficacy in this population do not mirror those reported from *S. mansoni* animal model studies [14, 15, 17]. In immunodeficient mice (nude, T cell depleted and severe combined immunodeficiency [SCID] mice), characterised by an absence of CD4^+^ T-lymphocytes and egg antibodies responses, praziquantel is inefficient in treating the *S. mansoni* infection [14, 15, 17]. However, it is important to note that the degree of immunosuppression in the mice used in these model studies was much greater than the moderate levels of CD4^+^ reduction found in the co-infected individuals in the present study. Interestingly, the passive transfer of serum from infected immunocompetent mice or from mice immunisation with *S. mansoni* worm antigens partly restores the efficacy of praziquantel in severely immunosuppressed mice [17, 19, 20], and this is dependent on specific anti-worm antibodies [19, 36].

In schistosomiasis endemic areas, young children are highly susceptible to infection and re-infection. However, an acquired partial immunity against infection [37] develops with age, with exposure to adult worms dying over time or following praziquantel treatment [19, 36]. In these endemic areas, most people will have acquired schistosomiasis very early in childhood and will already have had high levels of anti-worm antibodies by the time they were old enough to be included in this study. If their anti-worm antibodies remained high enough, despite the low to moderate reductions in CD4^+^ T cell counts that resulted from the HIV-1 infection, it is possible that these antibodies might contribute to the effective killing of the parasite by praziquantel, despite the immunosuppressive effects of the HIV-1 infection. It is likely that the moderate CD4^+^ T cell levels observed in co-infected individuals in the present study were sufficient to support the levels of anti-schistosome antibodies necessary for an enhanced praziquantel efficacy [19, 22, 36]. The CD4^+^ T cell levels that may be required to ensure an optimum praziquantel efficacy are not known. This calls for further, more detailed studies.

## Conclusion

Although this study was limited by the relatively low percentage of individuals who were co-infected with schistosomiasis and HIV-1, the results indicate that HIV-1 infection *per se* or its moderate immunosuppressive effects, as indicated by the CD4^+^ T cell counts, does not significantly affect praziquantel efficacy in terms of cure rate or reduction in the intensity of infection. This suggests that, in national schistosomiasis control programmes, the standard praziquantel dose can be effective, even deployed in populations where schistosomiasis and HIV-1 co-infections are likely to occur. This would also be the case for individual treatment except where there is an evidence of more severe HIV-1 or AIDS associated immunosuppression.

### Summary

The present study assessed the efficacy of praziquantel by comparing parasitological cure rates and the reduction in infection intensity in HIV-1 seronegative individuals infected with *S. mansoni* and HIV-1 seropositive individuals co-infected with *S. mansoni,* following treatment with a single oral dose of praziquantel.

## Electronic supplementary material

Additional file 1: **Multilingual abstracts in the six official working languages of the United Nations.** (PDF 286 KB)

## References

[1] Cioli D, Pica-Mattoccia L (2003). Praziquantel. Parasitol Res.

[2] WHO (2002). Prevention and control of schistosomiasis and soil-transmitted helminthiasis. World Health Organ Tech Rep Ser.

[3] Magnussen P (2003). Treatment and re-treatment strategies for schistosomiasis control in different epidemiological settings: a review of 10 years’ experiences. Acta Trop.

[4] Frohberg H (1984). Results of toxicological studies on praziquantel. Arzneimittelforschung.

[5] Bergquist NR (2002). Schistosomiasis: from risk assessment to control. Trends Parasitol.

[6] Fenwick A, Savioli L, Engels D, Robert Bergquist N, Todd MH (2003). Drugs for the control of parasitic diseases: current status and development in schistosomiasis. Trends Parasitol.

[7] Danso-Appiah A, De Vlas SJ (2002). Interpreting low praziquantel cure rates of Schistosoma mansoni infections in Senegal. Trends Parasitol.

[8] Gryseels B, Nkulikyinka L, Coosemans MH (1987). Field trials of praziquantel and oxamniquine for the treatment of schistosomiasis mansoni in Burundi. Trans R Soc Trop Med Hyg.

[9] Gryseels B, Mbaye A, De Vlas SJ, Stelma FF, Guisse F, Van Lieshout L, Faye D, Diop M, Ly A, Tchuem-Tchuente LA, Engels D, Polman K (2001). Are poor responses to praziquantel for the treatment of Schistosoma mansoni infections in Senegal due to resistance? An overview of the evidence. Trop Med Int Health.

[10] Stelma FF, Talla I, Polman K, Niang M, Sturrock RF, Deelder AM, Gryseels B (1993). Epidemiology of Schistosoma mansoni infection in a recently exposed community in northern Senegal. Am J Trop Med Hyg.

[11] Stelma FF, Talla I, Sow S, Kongs A, Niang M, Polman K, Deelder AM, Gryseels B (1995). Efficacy and side effects of praziquantel in an epidemic focus of Schistosoma mansoni. Am J Trop Med Hyg.

[12] Kumar V, Gryseels B (1994). Use of praziquantel against schistosomiasis: a review of current status. Int J Antimicrob Agents.

[13] Davis A, Jordan P, Webbe G, Sturrock RF (1993). Antischistosomal drugs and clinical practice. Human schistosomiasis.

[14] Sabah AA, Fletcher C, Webbe G, Doenhoff MJ (1986). Schistosoma mansoni: chemotherapy of infections of different ages. Exp Parasitol.

[15] Modha J, Lambertucci JR, Doenhoff MJ, McLaren DJ (1990). Immune dependence of schistosomicidal chemotherapy: an ultrastructural study of Schistosoma mansoni adult worms exposed to praziquantel and immune serum in vivo. Parasite Immunol.

[16] Doenhoff MJ, Bain J (1978). The immune-dependence of schistosomicidal chemotherapy: relative lack of efficacy of an antimonial in Schistosoma mansoni-infected mice deprived of their T-cells and the demonstration of drug-antiserum synergy. Clinical and Experimental Immunollogy.

[17] Doenhoff MJ, Sabah AA, Fletcher C, Webbe G, Bain J (1987). Evidence for an immune-dependent action of praziquantel on Schistosoma mansoni in mice. Trans R Soc Trop Med Hyg.

[18] Doenhoff MJ, Pearson S, Dunne DW, Bickle Q, Lucas S, Bain J, Musallam R, Hassounah O (1981). Immunological control of hepatotoxicity and parasite egg excretion in Schistosoma mansoni infections: stage specificity of the reactivity of immune serum in T-cell deprived mice. Transaction of the Royal Society of Tropical Medicine and Hygiene.

[19] Brindley PJ, Sher A (1987). The chemotherapeutic effect of praziquantel against Schistosoma mansoni is dependent on host antibody response. J Immunol.

[20] Doenhoff MJ, Modha J, Lambertucci JR (1988). Anti-schistosome chemotherapy enhanced by antibodies specific for a parasite esterase. Immunology.

[21] Secor WE, Sundstrom JB (2007). Below the belt: new insights into potential complications of HIV-1/schistosome coinfections. Curr Opin Infect Dis.

[22] Karanja DM, Boyer AE, Strand M, Colley DG, Nahlen BL, Ouma JH, Secor WE (1998). Studies on schistosomiasis in western Kenya: II. Efficacy of praziquantel for treatment of schistosomiasis in persons coinfected with human immunodeficiency virus-1. Am J Trop Med Hyg.

[23] Kallestrup P, Zinyama R, Gomo E, Butterworth AE, van Dam GJ, Gerstoft J, Erikstrup C, Ullum H (2006). Schistosomiasis and HIV in rural Zimbabwe: efficacy of treatment of schistosomiasis in individuals with HIV coinfection. Clin Infect Dis.

[24] Doenhoff MJ, Kusel JR, Coles GC, Cioli D (2002). Resistance of Schistosoma mansoni to praziquantel: is there a problem?. Trans R Soc Trop Med Hyg.

[25] Renganathan E, Cioli D (1998). An international initiative on praziquantel use. Parasitol Today.

[26] WHO (1990). Report of the WHO Informal Consultation on Monitoring of Drug Efficacy in the Control of Schistosomiasis and Intestinal Nematodes.

[27] Malenganisho WLM (2005). The Role of HIV, Micronutrient Status and Treatment in Schistosoma Mansoni Infection and Morbidity: a Cohort Study Among Adult of Ukerewe and Mwanza districts, Tanzania. PhD thesis.

[28] Malenganisho WL, Magnussen P, Friis H, Siza J, Kaatano G, Temu M, Vennervald BJ (2008). Schistosoma mansoni morbidity among adults in two villages along Lake Victoria shores in Mwanza District, Tanzania. Transaction of the Royal Society of Tropical Medicine and Hygiene.

[29] Magendantz M (1972). The biology of Biomphalaria choanomphala and B. sudanica in relation to their role in the transmission of Schistosoma mansoni in Lake Victoria at Mwanza, Tanzania. Bullettin of the World Health Organization.

[30] Kardorff R, Gabone RM, Mugashe C, Obiga D, Ramarokoto CE, Mahlert C, Spannbrucker N, Lang A, Gunzler V, Gryseels B, Ehrich JH, Doehring E (1997). Schistosoma mansoni-related morbidity on Ukerewe Island, Tanzania: clinical, ultrasonographical and biochemical parameters. Trop Med Int Health.

[31] Pickering H, Okongo M, Bwanika K, Nnalusiba B, Whitworth J (1997). Sexual behaviour in a fishing community on Lake Victoria, Uganda. Health Transit Rev.

[32] Katz N, Chaves A, Pellegrino J (1972). A simple device for quantitative stool thick-smear technique in Schistosomiasis mansoni. Revista Instituto de Medicine Tropica Sao Paulo.

[33] United republic of Tanzania Ministry of Health and Social Welfare National Aids Control Programme (2007). Guidelines for HIV Testing and Counseling in Clinical Settings.

[34] WHO (2007). Guidance on Provider-Initiated HIV Testing and Counseling in Health Facilities.

[35] Utzinger J, N’Goran EK, N’Dri A, Lengeler C, Tanner M (2000). Efficacy of praziquantel against Schistosoma mansoni with particular consideration for intensity of infection. Trop Med Int Health.

[36] Harnett W, Kusel JR (1986). Increased exposure of parasite antigens at the surface of adult male Schistosoma mansoni exposed to praziquantel in vitro. Parasitology.

[37] Butterworth AE (1998). Immunological aspects of human schistosomiasis. Br Med Bull.

