# Supplementary material for: Praziquantel efficacy against Schistosoma mansoni among HIV-1 infected and uninfected adults living in fishing villages along Lake Victoria, Northwest Tanzania
Source: Infect Dis Poverty. 2014 Dec 15;3:47. doi: 10.1186/2049-9957-3-47 (PMC4322465; doi:10.1186/2049-9957-3-47)
Supplement: Supplementary file 1 — Additional file 1: Multilingual abstracts in the six official working languages of the United Nations. (PDF 286 KB) [file 40249_2014_87_MOESM1_ESM.pdf]

Translation of the abstract into the six official working languages of the United Nations

فاعلية البرازيكوانتيل ضد بين البالغين المصابين وغير المصابين بفيروس HIV-1 الذين يعيشون بقرى الصيد على امتداد بحيرة فيكتوريا، شمال غرب تنزانيا

همفري د. مزيجو، ديفيد و. دان، سفاري م. كينانجاهي، فريد نواها

ملخص

معلومات عامة: أظهرت الدراسات على الحيوانات أن الاستجابات المناعية الوظيفية، كما تحددها مستويات تعداد خلايا $CD4^+$ واستجابات الأجسام المضادة لمضاد البلهارسية، هي التي تحدد فاعلية البرازيكوانتيل. بناءً على هذا الدليل تم وضع افتراض أن آثار العوز المناعي للإصابة بفيروس العوز المناعي البشري 1-(HIV) يمكن أن تؤثر على فاعلية البرازيكوانتيل لدى المضيف البشري المصاب بعدوى مرافقة. وعلى هذا النحو، تعمل الدراسة الحالة على تقييم فاعلية البرازيكوانتيل عن طريق مقارنة معدلات الشفاء من الطفيليات وانخفاض كثافة الإصابة بالعدوى في أفراد سلبيي المصل لفيروس HIV-1 مصابين وأفراد إيجابيي المصل لفيروس HIV-1 مصابين بعدوى مرافقة بعد العلاج بجرعة واحدة بالفم من البرازيكوانتيل.

المناهج: يمثل هذا المنهج دراسة طولية مستقبلية اشتملت أساساً على 555 فرداً بالغاً مصاباً تتراوح أعمارهم بين 21-55 عاماً سواء من أولئك المصابين أو غير المصابين بعدوى مرافقة بفيروس HIV-1 ويعيشون بقرى الصيد على امتداد بحيرة فيكتوريا بشمال غرب تنزانيا. تم علاج هؤلاء الأفراد بجرعة واحدة بالفم من البرازيكوانتيل (40 مجم/كجم)، وتم أخذ عينات براز في الأسبوع الثاني عشر لفحص وجود بيض باستخدام تقنية كاتو-كاتز. كما تم جمع عينات وخز الإصبع وسحب الدم الوريدي لفحص فيروس HIV-1 وتعداد خلايا $CD4^+$.

النتائج: لم يختلف معدل الشفاء من الطفيليات إلى حد كبير عن الحالة المصلية لفيروس HIV-1، (P=0.12): بلغ معدل الشفاء بين الأفراد المصابين بالعدوى المرافقة 48.3% (14/29)، أما بين الأفراد المصابين فقط فقد بلغ معدل الشفاء 62.6% (329/526). لم تختلف نسبة انخفاض البيض عن الحالة المصلية لفيروس HIV-1، (P=0.22): فبلغت 77.22% في الأفراد سلبيي المصل لفيروس HIV-1 و75% في الأفراد إيجابيي المصل لفيروس HIV-1. لم يؤثر مستوى تعداد خلايا $CD4^+$ (وسيط 228 خلية/ميكرولتر: مدى 202-380 خلية) على معدل الشفاء (P=0.23) أو على انخفاض كثافة الإصابة بالعدوى (P=0.37).

الاستنتاج: لم تؤثر عدوى HIV-1 في حد ذاتها أو الآثار المعتدلة للعوز المناعي، التي أظهرها مدى تعداد خلايا $CD4^+$ الملاحظ في الأفراد المصابين بالعدوى المرافقة، على فاعلية البرازيكوانتيل وفقاً لقياسات الدراسة الأترابية الحالية لمعدلات الشفاء من الطفيليات وانخفاض كثافة الإصابة بالعدوى.

Translated from English version into Arabic by Ran Abdel Rahman, through

TRANSLATORS
WITHOUT BORDERS

坦桑尼亚西北部维多利亚湖附近渔村 HIV 感染者和非感染者中，吡喹酮治疗曼氏血吸虫效果观察

Humphrey D. Mazigo, David W. Dunne, Safari M. Kinung’hi, Fred Nuwaha

摘要

引言：动物研究显示，与 $CD4^+$细胞数量及抗血吸虫抗体反应相关的免疫功能决定了吡喹酮的疗效。由此我们认为 HIV 感染造成的免疫功能缺陷将会影响吡喹酮对合并 HIV 感染的血吸虫感染者的疗效。因此我们开展此项研究，通过比较两组人群（血吸虫合并和未合并 HIV 感染）在接受一次口服剂量的吡喹酮治疗后的治愈率和减虫率，评估吡喹酮对血吸虫合并 HIV 感染人群的治疗效果。

方法：采用前瞻性队列研究方法，在坦桑尼亚西北部的维多利亚湖周围的渔村调查了 555 名年龄在 21-55 岁之间的曼氏血吸虫感染者，采集静脉血进行 HIV 和 $CD4^+$细胞计数检测。根据是否合并 HIV 感染分为实验组和对照组，两组人群均给予一次性口服剂量的吡喹酮治疗（(40 mg/kg)，在第 12 周，采集两组人群的粪便样本，采用改良加藤法检测曼氏血吸虫卵。

结果：曼氏血吸虫合并 HIV 感染者服用吡喹酮的治愈率和减虫率分别为 48.3%(14/29)和 75%，曼氏血吸虫未合并 HIV 感染者服用吡喹酮的治愈率和减虫率分别为 62.6% (329/526)和 77.22%，两组之间的治愈率和减虫率的差别均无无统计学意义（*P* 值分别为 0.12 和 0.22）。感染者 $CD4^+$细胞计数水平（中位数为 228 个/μL（202-380 个/μL））对治愈率和减虫率的影响也无统计学意义（*P* 值分别为 0.23 和 0.37）。

结论：HIV 感染本身或由于 HIV 感染引起的免疫功能改变（$CD4^+$细胞计数的变化）对吡喹酮治疗曼氏血吸虫的治愈率和减虫率的效果无显著影响。

Translated from English version into Chinese by Tian Li-guang, through

CHINESE CENTER FOR DISEASE CONTROL AND PREVENTION
NATIONAL INSTITUTE OF PARASITIC DISEASES

Efficacité du Praziquantel par rapport au *Schistosoma mansoni* chez les adultes infectés et non affectés par le VIH-1 vivant dans les villages de pêcheurs aux rives du lac Victoria, au nord-ouest de la Tanzanie.

Humphrey D. Mazigo, David W. Dunne, Safari M. Kinung’hi, Fred Nuwaha

Résumé

Contexte : Des études chez les animaux ont démontré que les réponses immunitaires fonctionnelles, déterminées par les niveaux de comptages des cellules $CD4^+$ et les réactions des anticorps anti-schistosomes, déterminent l’efficacité du praziquantel. En se basant sur cette preuve, une hypothèse a été émise que les effets de l’infection du virus de l’immunodéficience humaine (VIH-1) peuvent affecter l’efficacité du praziquantel chez les hôtes humaines infectées. Ainsi, la présente étude a evalué l’efficacité du praziquantel en comparant les taux de guérison parasitologiques et la diminution de l’intensité de l’infection chez les individus séropositifs VIH-1 infectés par *S. mansoni* et les individus séropositifs HIV-1 co-infectés par *S. mansoni,* suite à un traitement avec une seule dose de praziquantel.

Méthodes : C’était une étude longitudinale prospective qui comprenait, au départ, 555 adultes infectés du *S. mansoni* âgés de 21-25 ans, qui étaient co-infectés ou non par le VIH-1 et qui vivaient dans des villages de pêcheurs sur les rives du lac Victoria au nord-ouest de la Tanzanie. Ces personnes ont été traitées avec une seule dose de praziquantel (40 mg/kg) et, après 12 semaines, des échantillons de selles ont été prélevés et examinés pour

des œufs *S. mansoni* en utilisant la technique Kato-Katz. Des échantillons de sang par piqûres au doigt et veineux ont été prélevés pour le dépistage du VIH-1 et la quantification cellulaire de CD4^{+}.

Résultats : Le taux de guérison parasitologique ne différait pas significativement de la séropositivité VIH-1 (*P*=0.12): parmi les individus co-infectés, le taux de guérison était de 48.3% (14/29), et parmi les personnes infectées uniquement de *S. mansoni*, le taux de guérison était de 62,6% (329/526). Le taux de réduction d'œufs n'a pas varié du statut sérologique du VIH-1 (*P*=0.22): 77.22% pour séropositifs VIH-1 et 75% pour les individus séropositifs VIH-1. Le niveau du comptage de cellules CD4^{+} (cellules médianes 228 /µL: variation de 202–380 cellules) n'a pas influencé le taux de guérison (*P*=0.23) ou la réduction de l'intensité de l'infection (*P*=0.37).

Conclusion : l'infection VIH-1 *per se* ou ses effets modérés d'immunodéficience, démontrée par la variation de comptages de cellules CD4^{+} chez les individus co-infectés, n'a pas affecté l'efficacité du praziquantel, telle mesurée par le taux de guérison parasitologique et la réduction de l'intensité de l'infection dans la présente cohorte de l'étude.

Translated from English version into French by ishaklamia, through

TRANSLATORS WITHOUT BORDERS

Эффективность празиквантела для лечения *Schistosoma mansoni* среди ВИЧ-инфицированного и не-ВИЧ-инфицированного взрослого населения рыбацких поселков на озере Виктория в северо-восточной Танзании.

Хамфри Д. Мазиго, Давид В. Данн, Сафари М. Кинунги, Фред Нуваха (Humphrey D. Mazigo, David W. Dunne, Safari M. Kinung'hi, Fred Nuwaha)

Синопсис

Справочная информация: Исследования на животных показали, что функциональные иммунные реакции, определяемые количеством CD4^{+} клеток и реакциями на анти-шистозомные антитела, определяют эффективность празиквантела. На основании этих данных было предложено, что эффект иммунодефицита при ВИЧ-1 инфекции может оказать негативное влияние на эффективность празиквантела на ко-инфицированных человеческих хозяевах. Таким образом, в данном исследовании оценивается эффективность празиквантела путем сравнения паразитологических показателей эффективности лечения и уменьшение интенсивности инфекции среди ВИЧ-1 инфицированных серонегативных пациентов с *S. mansoni* и ВИЧ-1 инфицированных серопозитивных пациентов, ко-инфицированных *S. mansoni,* после терапии с применением однократной оральной дозы празиквантела.

Методология: Это было проспективное лонгитюдное исследование, в котором приняли участие 555 взрослых возрастом от 21 до 55 лет, инфицированных *S. mansoni*, которые также были или не были ко-инфицированы ВИЧ-1, которые проживали в рыбацких поселках на берегу озера Виктория. Была проведена терапия одной дозой празиквантела (40 мг/кг) и через 12 недель были взяты и исследованы на наличие яиц *S. mansoni* образцы кала, используя метод Като-Кац. Образцы венозной крови и крови из пальца были отобраны для скрининга ВИЧ-1 инфекции и подсчета клеток CD4^{+}.

Результаты: Паразитологические показатели эффективности лечения существенно не отличаются по ВИЧ-1 серологическому статусу (*P*=0.12): среди ко-инфицированных пациентов показатели

эффективности лечения составили 48.3% (14/29), и среди пациентов, инфицированных только *S. mansoni*, показатель эффективности составил 62.6% (329/526). Уменьшение количества яиц не зависело от ВИЧ-1 серологического статуса (*P*=0.22): 77.22% среди ВИЧ-1 серонегативных пациентов и 75% среди ВИЧ-1 серопозитивных. Количество клеток CD4$^+$ (средний показатель 228 клеток/мкл: в диапазоне 202–380 клеток) не влияло на эффективность лечения (*P*=0.23) или на уменьшение интенсивности инфекции (*P*=0.37).

Заключение: Наблюдаемые количества клеток CD4$^+$ среди ко-инфицированных пациентов свидетельствует о том, что ВИЧ-1 инфекция как таковая, включая умеренный эффект на иммунодефицит, не влияет на эффективность празиквантела при измерении паразитологической эффективности лечения и уменьшения интенсивности инфекции в исследуемой группе пациентов.

Translated from English version into Russian by Julia Zarubinska-Toepritz, through

TRANSLATORS
WITHOUT BORDERS

Eficacia del praziquantel (PZQ) contra la esquistosomiasis mansoni entre infectados por el VIH-1 y adultos no infectados que viven en aldeas de pescadores a orillas del Lago Victoria, al noroeste de Tanzania

Humphrey D. Mazigo, David W. Dunne, Safari M. Kinung'hi, Fred Nuwaha

Resumen

Antecedentes: Estudios en animales han demostrado que las respuestas inmunes funcionales, y su relación con el conteo de células CD4$^+$ y las respuestas de los anticuerpos anti-esquistosomiasis, determinan la eficacia de praziquantel. Basándose en esta evidencia, se plantea como hipótesis que los efectos de inmunodeficiencia de la infección del virus de inmunodeficiencia humana (VIH-1) pueden afectar a la eficacia de praziquantel en huéspedes humanos coinfectados. Así pues, el presente estudio evaluó la eficacia de praziquantel comparando la tasa de curación parasitológica y la reducción de la intensidad de las infecciones por VIH-1 en individuos seronegativos infectados con *S. mansoni* y seropositivos de VIH-1 coinfectados con *S. mansoni, y* continuando el tratamiento con una dosis única oral de praziquantel.

Métodos: Este fue un estudio longitudinal prospectivo que incluyó, en el momento de referencia, 555 adultos infectados con S. *mansoni* de edades comprendidas entre los 21 y 55 años, coinfectados o no con el VIH-1 y que vivían en las aldeas de pescadores a orillas del Lago Victoria en el Noroeste Tanzania. Estas personas fueron tratadas con una dosis única oral de praziquantel (40 mg/kg) y a las 12 semanas, se obtuvieron muestras de heces individuales y se examinaron utilizando la técnica de Kato-Katz para comprobar la existencia de huevos *de S. mansoni*. Se llevaron a cabo tests de punción del dedo y se tomaron muestras de sangre venosa para la detección de VIH-1 y la cuantificación de células CD4$^+$.

Resultados: La tasa de curación parasitológica no difiere significativamente entre los estados serológicos de VIH-1 (*P*=0.12): entre los coinfectados, la tasa de curación fue del 48.3 % (14/29), y entre los individuos infectados sólo con *S. mansoni* , la tasa de curación fue del 62.6 % (329/526). La tasa de reducción del número de huevos no varía con el estado serológico VIH-1 (*P*=0.22): 77.22 % para seronegativos VIH-1 y 75% para

seropositivos de VIH-1. El conteo de células $CD4^+$ (mediana de 228 células/μL: rango 202-380 células) no influyó en la tasa de curación (P=0.23) o la disminución de la intensidad de la infección (P=0.37).

Conclusión: La infección por el VIH-1 *per se o* sus efectos de inmunodeficiencia moderada, como se demuestra en los conteos de la gama de células $CD4^+$ observada en las personas infectadas, no afectan a la eficacia de praziquantel, como se demostró en la tasa de curación parasitológica y la reducción de la intensidad de la infección en el presente estudio cohorte.

Translated from English version into Spanish by Sonsoles Marín, through

TRANSLATORS
WITHOUT BORDERS
